# Protective Effect of Grape (*Vitis vinifera*) Seed Powder and Zinc-Glycine Complex on Growth Traits and Gut Health of Broilers Following *Eimeria tenella* Challenge

**DOI:** 10.3390/antibiotics10020186

**Published:** 2021-02-14

**Authors:** Naila Chand, Pervez Ali, Ibrahim A. Alhidary, Mutassim A. Abdelrahman, Hani Albadani, Murad Ali Khan, Alireza Seidavi, Vito Laudadio, Vincenzo Tufarelli, Rifat Ullah Khan

**Affiliations:** 1 Department of Poultry Science, Faculty of Animal Husbandry & Veterinary Sciences, The University of Agriculture Peshawar, Peshawar 25000, Pakistan; naila@gmail.com (N.C.); pervezali@gmail.com (P.A.); 2 Department of Animal Production, College of Food and Agriculture Science, King Saud University, Riadh 96400, Saudi Arabia; ialhidary@ksu.edu.sa (I.A.A.); amutassim@ksu.edu.sa (M.A.A.); halbadani@ksu.edu.sa (H.A.); rukhan@aup.edu.pk (R.U.K.); 3 College of Veterinary Sciences, Faculty of Animal Husbandry & Veterinary Sciences, The University of Agriculture Peshawar, Peshawar 25000, Pakistan; drmurad@gmail.com; 4 Department of Animal Science, Rasht Branch, Islamic Azad University, Rasht 41335-3516, Iran; alirezaseidavi@iaurasht.ac.ir; 5 Department of DETO, Section of Veterinary Science and Animal Production, University of Bari ‘Aldo Moro’, Valenzano, 70010 Bari, Italy; vito.laudadio@uniba.it

**Keywords:** broiler, grape seeds, organic zinc, coccidiosis, growth

## Abstract

The current study was conducted to evaluate the impact of grape (*Vitis vinifera*) seed powder and zinc-glycine complex on growth parameters and cecal health of broiler following *Eimeria tenella* challenge. A total of 250 day-old male broilers (Hubbard) were distributed into five treatments as follows: I—negative control (basal diet); II—positive control (*E. tenella* challenge); III—group infected + Amprolium; IV—group coccidial infection + organic zinc (OZ) at 50 ppm + grape seed powder (GSP) at 2.5 g/kg; V—coccidial infection + organic zinc (OZ) at 50 ppm + grape seed powder (GSP) at 5.0 g/kg. From findings, a lower (*p* < 0.05) feed intake was noted for positive control compared to the other treatment groups. Conversely, higher (*p* < 0.05) body weight, dressing percentage, and feed conversion ratio were observed in infected + Amprolium, GSP2.5 + OZ50, and GSP5 + OZ50 treatments in comparison to the positive control. Moreover, the positive control showed severe cecal lesions of thickness and hemorrhages with mild congestion. The lesion scores decreased (*p* < 0.05) in GSP and OZ treated groups compared to the positive control. Significant (*p* < 0.05) lower oocyst per gram of feces was found in infected + Amprolium, GSP2.5 + OZ50, and GSP5 + OZ50 in comparison to positive control. Supplementing diet with GSP and OZ at both levels showed restoration of intense sloughing of villi. From the present findings, it can be concluded that OZ and grape seed powder positively ameliorated the growth performance, lesion score, and oocysts shedding in broilers infected with *E. tenella*.

## 1. Introduction

Coccidiosis is one of the deadliest enteric diseases, causing severe intestinal damage and stunt growth in broilers [1]. This disease is primarily controlled by using chemotherapeutics, mainly anticoccidial drugs [2]. However, the extensive use of anticoccidial drugs has reportedly resulted in the development of resistance against *Eimeria* [3]. The condition is further exacerbated by public concerns over drug residues in poultry products. The search for alternatives to these anticoccidial drugs has resulted in an exhaustive list of feed additives of diverse nature [4,5]. These feed additives improve the immune system and reduce the stress level leading to enhanced performance during coccidial infection in birds.

Zinc (Zn) is a biologically important trace mineral and is involved as a cofactor in the metabolic functions of almost 300 enzymes in the body [6]. Recent studies have shown that compared to inorganic Zn, organic Zn improved the immune system response against the coccidiosis, lessened lesion scores, and heightened performance in broilers [7,8,9]. During coccidial infection, Zn absorption is further decreased in the upper part of the intestines [9]. In the past, different studies have identified different kinds of chelates or complexes of organic Zn; however, there is a continuous effort to identify other forms of organic Zn complexes that show better bioavailability, stability, and physical homogeneity, such as Zn-glycine (Zn-Gly) complex [8,10,11]. The use of Zn-Gly chelate has shown greater beneficial impacts on the growth and immune responses, intestinal features, and antioxidant enzymes activities in broilers [8,11]. During coccidial challenge, Zn metabolism is highly decreased; therefore, there is an imperative need of supplementation of Zn in the diet [12].

Grape (*Vitis vinifera*) is grown as one of the largest fruit crops worldwide. Grapes are a good source of flavonoids and phenols and can be easily incorporated in animals’ diets. Some of the flavonoids of grape extract, such as catechins, are absorbed in the small intestines, while other polymers that escape absorption reach the colon and are metabolized into phenolic acids by the microbiome [13]. Different forms of grape such as extract, pomace, or seed proanthocyanidin resulted in less mortality and lesion score of *Eimeria tenella* infection in broilers [14]. A recent study has shown that 2% grape seed extract improved metabolism through increasing the population of useful enteric bacteria such as *Lachnospiraceae, Lactobacilliaceae*, and *Bifidobacteriaceae* in breeder hens [13]. To the best of our knowledge, no study has reported the effect of grape seeds powder (GSP) in coccidial infection in broilers; in addition, most of the studies on the use of organic Zn against coccidial infection are inconclusive. Therefore, the aim of the present study was to evaluate the effect of organic Zn-Gly and GSP on the growth performance, oocysts shedding, and cecal lesions in broilers infected with coccidiosis.

## 2. Materials and Methods

### 2.1. Animals

A total of 250 one-day old male broilers (Hubbard) were obtained from a local hatchery and individually weighed. Birds were distributed into five treatments having five replicates. A paper sheet was placed to collect excreta and changed daily. All birds were free from coccidial infection and anticoccidial vaccine. Birds in treatment one (negative control) were fed a basal diet with no coccidial challenge; the 2nd group (positive group) was infected with *E. tenella* challenge; the 3rd group (infected + Amprolium) was challenged with *E. tenella* infection and supplemented with Amprolium (1 g/kg; Huvepharma Inc., Peachtree City, GA, USA); the 4th group was challenged with *E. tenella* infection and supplemented with organic zinc (OZ, 50 ppm) and grape seed powder (GSP, 2.5 g/kg; GSP2.5 + OZ50); the 5th group was challenged with *E. tenella* infection and supplemented with organic zinc (50 ppm) and grape seed powder (5 g/kg; GSP5 + OZ50). Chicks were initially brooded at a temperature above 30 °C for the first week until the temperature was brought to 22–23 °C in the following week. Birds were exposed to a fluorescent lighting program for 23 h (21 Lux) per day. Corn- and soybean-based diets were formulated on the basis of starter and finisher phases (Table 1) without any antibiotic addition and supplied ad libitum with free access to water to all the birds.

### 2.2. Coccidial Challenge

On day 8, all treatments were inoculated with 5 × 10^4^ sporulated oocysts of *E. tenella* by oral gavage except the noninfected control (negative control). Mortality and excreta conditions were recorded on a daily basis after the infection. The *E. tenella* strain used in this study was provided courteously by the Veterinary Research Institute, Peshawar.

### 2.3. Performance Traits and Sampling

Feed intake and body weight were recorded on a cage basis (10 birds). Feed conversion ratio (FCR) was calculated on the obtained data of feed intake and body weight gain. On day 35, two birds per replicate were randomly selected and killed by cervical dislocation. The feathers, head, feet, and internal organs were removed, and dressing percentage was calculated.

### 2.4. Lesion Score

Lesion score of the ceca of two birds per replicate was determined comprised of thickness, hemorrhages, and congestion in ceca. The lesion score was numerated as suggested by Ali et al. [3] as: 0 = No lesion, + = Mild lesion, ++ = Moderate lesion, +++ = Severe lesion.

### 2.5. Number of Oocysts per Gram (OPG)

For oocysts number, total fecal output of each cage at 7, 14, and 21 days post-infection (dpi) was determined by the method as described by Ali et al. [3]. Concentrated sodium chloride solution containing oocysts was poured in a McMaster chamber and the numbers of oocysts were counted with the help of a microscope.

### 2.6. Histopathological Examination

Further cecal samples were aseptically separated and processed for histopathological examination by the method of Chand et al. (2016). About 1 cm cecal tissue was separated and passed in 10% buffer formalin, followed by dehydration by graded levels of alcohol solution. Tissues blocks were sectioned using microtome (Accu-Cut SRM 200 Sakura, Finetek, Holland). Slides were stained and viewed under a microscope.

### 2.7. Statistical Analysis

Data were statistically analyzed through analysis of variance (ANOVA) by using a randomized complete block design (RCBD). Means were compared for significance difference by least significant difference (LSD). Statistical package (STATISTIC-2010) was used to carry out above analysis.

## 3. Results

The total and weekly feed consumption of broilers were affected by the combination of GSP and OZ as in Table 2. During the 3rd and 5th weeks, the maximum and the same feed intake was observed in the uninfected control group, infected + Amprolium, GSP2.5 + OZ50, and GSP5 + OZ50; and less (*p* < 0.05) feed intake was noted in the positive control. During the 4th week, significantly (*p* < 0.05) higher and the same feed was observed in the negative control group, infected + Amprolium, GSP2.5 + OZ50, and GSP5 + OZ50, while less (*p* < 0.05) feed intake was noted for the positive control. Lower total feed intake was found in the positive control group, while maximum and non-significantly (*p* > 0.05) the same feed intake was noted for the negative control, infected + Amprolium, GSP2.5 + OZ50, and GSP5 + OZ50 groups. The results of total and weekly mean body weight gain, except for the 2nd week, were affected significantly (*p* < 0.05) by the supplementation of GSP and OZ (Table 3). During the 3rd week, maximum weight gain was noted in the negative control and then by infected + Amprolium, GSP2.5 + OZ50, and GSP5 + OZ50, while less feed intake was recorded for the positive control. In the 5th week and overall basis, body weight was maximum in the negative control followed by groups of infected + Amprolium, GSP2.5 + OZ50, and GSP5 + OZ50, while significantly (*p* < 0.05) lower weight gain was noted for the positive control.

The overall and weekly FCR of birds is reported in Table 4. Supplementation of GSP and OZ affected FCR of *E. tenella* challenged broilers at all periods except during the 2nd week of the trail. During the 4th week, minimum and the same FCR was observed for uninfected untreated and infected + Amprolium and was followed by GSP2.5 + OZ50 and GSP5 + OZ50. Total FCR was the maximum for the positive control, while minimum FCR was recorded for negative control. The effect of GSP and OZ significantly (*p* < 0.05) affected the dressing percentage of broilers infected with *E. tenella* (Table 5). Significantly higher (*p* < 0.05) dressing percentage was noted for the negative control group and infected +Amprolium as compared to the positive control.

As shown in Table 6, dietary supplementation of GSP and OZ significantly (*p* < 0.05) affected OPG of feces in broilers on 7, 14, and 21 dpi. Among the challenged groups, at 7 dpi the feces sample of infected untreated group had maximum oocysts count of *E. tenella* followed by GPS and OZ supplemented groups, while the lowest number of oocysts were counted in infected + Amprolium. At 14 and 21 dpi, the maximum oocysts were recorded for the positive control and then for GSP and OZ treated birds.

**Figure 1:**
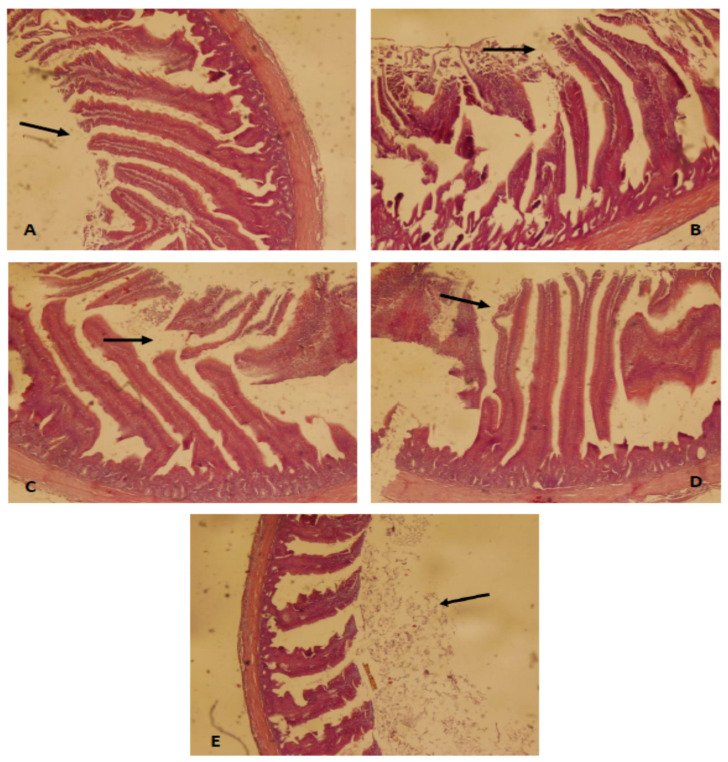
Photomicrograph (40×) of cecum from: negative control (**A**) with arrow showing a normal epithelium; positive control (**B**) with arrow showing broken epithelium; Amprolium treated (**C**) with arrow showing regenerating epithelium; GSP2.5 + OZ50 (**D**) with arrow showing slightly broken epithelium but also the healing effect of the treatment; and GSP5 + OZ50 (**E**) with arrow showing improved epithelial layers in infected birds after the treatment.

Histopathological study of ceca for the positive control showed shorting of crypt, sloughing of villi, and glandular necrotic structure. Infected and supplemented groups with GSP and OZ at both levels showed mild sloughing of villi. Infected + Amprolium treated birds revealed normal cecal histology (Figure 1). The lesion score of birds infected with *E. tenella* in the control and treatment groups are given in Table 7. The positive control showed severe lesions of thickness and hemorrhages with mild congestion. The lesion scores of all parameters decreased in GSP and OZ treated groups.

## 4. Discussion

To the best of our knowledge, this is the first study reporting the combined effect of GSP and OZ in broilers during *E. tenella* infection. Previous studies have reported the individual effects of grape seed proanthocyanidins extract and organic Zn supplied individually [7,8,9,14,15]. In the current study, the growth performance was much improved in the infected birds supplemented with GSP and OZ. Grape seed powder is a valuable source of polyphenols, which act as powerful antioxidants and improve the immunity of birds and increase the population of beneficial bacteria in the ileum, and enhance villus height and crypt depth, which led to increased absorption of feed nutrients and digestion in broilers [15]. Zinc is necessary for all digestive functions of the avian-like secretion of digestive enzymes, thereby enhancing digestibility of nutrients and growth performance of birds [11]. The absorption of Zn is correlated with the type of *Eimeria* species. Since *E. tenella* invades the cecum, while Zn is absorbed in the duodenum of the intestines, it is speculated that Zn absorption by the chickens is not affected during *E. tenella* infection. This speculation was confirmed from the study of He et al. [9], who reported that blood Zn was partially restored by OZ supplementation in chickens infected with *Eimeria maxima* and *Clostridium perfringens*.

In the current study, oocysts shedding was significantly decreased in broilers treated with GPS and OZ. In a study, Rapp et al. [16] reported reduced gut lesion scores in response to Zn-amino acid complex in broilers infected with *E. maxima*. In another trial, Bun et al. [11] found reduced oocysts shedding in broilers treated with Zn-methionine chelate during *E. tenella* infection. The exact mechanism of how OZ reduces the oocysts population in the cecum is not completely known. It is speculated that OZ supports intestinal defense against coccidia by increasing the level of IgA [11,12], which decreases the infection by attaching directly to the coccidian surface and preventing their attachment to the gut epithelium [17]. Further, Wang et al. [14] suggested that GSPE at the level of 10–20 mg/kg reduced the oocysts shedding due to increasing plasma superoxide dismutase and decreasing oxidative stress and plasma nitric oxide, which collectively restored the oxidant–antioxidant balance disturbed by the *E. tenella* infection.

In the current study, the disturbed villus histology was slightly restored by the supplementation of OZ and GPS in the infected birds. Recent studies have shown several positive impacts of OZ on the intestinal microbiota, permeability of the immune system, and inflammation in broilers [12,18]. Recently, Bortoluzzi et al. [19] reviewed that OZ downregulated the inflammatory cytokines, upregulated the expression of A20 (an anti-inflammatory regulator), and promoted IgA and MUC2 production, which ultimately led to improved villus dimensions in broilers infected with *E. maxima*, as observed in the current study. Similarly, the addition of GSP imparted a positive effect on the intestinal health of chickens under infection of *E. tenella* in the current study. In a study, Viveros et al. [15] reported that poly-phenol-rich grapes enhanced villus dimensions in 21 day-old broiler chickens. Similar to our study, Yang et al. [20] suggested that grape proanthocyanidin at the rate of 7.5 and 15 mg/kg improved the jejunum morphology in broilers. Bagchi et al. [21] suggested that proanthocyanidin protects the intestinal mucosa from oxidative stress and inhibits pathogenic intestinal microflora, leading to healthy intestines.

## 5. Conclusions

From the results of the present study, it was concluded that organic Zn and grape seed powder positively ameliorated the negative effects on growth performance, lesion score, and oocysts shedding in broilers infected with *E. tenella*. In addition, doubling the dose of grape seed powder did not affect the parameters significantly, which needs further clarification.

## Figures and Tables

**Table 1:** Basal composition of diet fed to broilers during the starter and finisher phase.

Ingredients, %	Starter	Finisher
Corn	53.21	60.75
Soybean meal	37.92	25.00
Corn gluten meal	2.00	7.10
Corn oil	2.20	2.80
Dicalcium phosphate	2.30	2.05
Limestone	0.83	0.68
Salt	0.45	0.50
Vitamin–mineral premix ^1^	0.50	0.50
DL-Methionine	0.20	0.10
Lysine-HCl	0.22	0.37
Threonine	0.11	0.10
Choline chloride	0.05	0.05
**Chemical Composition**		
ME, kcal/kg	3000	3150
Crude protein, %	23.50	21.30
Methionine, %	0.55	0.44
Lysine, %	1.42	1.23
Sulfur amino acids, %	0.96	0.80
Threonine, %	0.95	0.85
Calcium, %	1.05	0.90
Phosphorus, %	0.50	0.45

^1^ Vitamin–mineral premix contains the following per kg: vitamin A, 2,400,000 IU; vitamin D, 1,000,000 IU; vitamin E, 16,000 IU; vitamin K, 800 mg; vitamin B1, 600 mg; vitamin B_2_, 1600 mg; vitamin B_6_, 1000 mg; vitamin B_12_, 6 mg; niacin, 8000 mg; folic acid, 400 mg; pantothenic acid, 3000 mg; biotin 40 mg; antioxidant, 3000 mg; cobalt, 80 mg; copper, 2000 mg; iodine, 400 mg; iron, 1200 mg; manganese, 18,000 mg; selenium, 60 mg; and zinc, 14,000 mg.

**Table 2:** Mean ± SE of feed consumption in grape seed powder and organic Zn fed broilers challenged with coccidiosis.

Groups	2nd Week	3rd Week	4th Week	5th Week	Overall Mean
Negative control	389.6 ± 8.22	614.2 ^a^ ± 16.10	750.5 ^a^ ± 71.1	920.67 ^a^ ± 7.94	2675.3 ^a^ ± 34.99
Positive control	391.4 ± 11.6	592.2 ^b^ ± 4.10	610.17 ^c^ ± 16.75	796.20 ^b^ ± 15.32	2400.7 ± 28.55
Infected + Amprolium	391.7 ± 12.1	617.47 ^a^ ± 4.89	722.07 ^b^ ± 13.34	890.73 ^a^ ± 37.85	2634.3 ^a^ ± 41.04
GSP2.5 + OZ50	387.8 ± 16.5	624.77 ^a^ ± 8.50	741.80 ^ab^ ± 18.04	912.17 ^a^ ± 5.00	2666.6 ^a^ ± 8.70
GSP5 + OZ50	389.2 ± 8.82	627.37 ^a^ ± 8.60	738.77 ^ab^ ± 10.11	918.57 ^a^ ± 11.45	2672.9 ^a^ ± 33.68
*p*-value	0.993	0.007	≤0.01	≤0.01	≤0.01

Mean values bearing different superscripts (a, b) in the column differ significantly (*p* < 0.05); GSP: grape seed powder; OZ: organic Zn.

**Table 3:** Mean ± SE of weight gain in grape seed powder and organic zinc supplemented broilers challenged with coccidiosis.

Groups	2nd Week	3rd Week	4th Week	5th Week	Overall Mean
Negative control	281.67 ± 3.51	392.33 ± 9.07	466.33 ^a^ ± 9.07	502.3 ^a^ ± 7.5	1642.7 ^a^ ± 15.17
Positive control	280.33 ± 5.50	353.67 ^d^ ± 7.09	287.33 ^d^ ± 7.09	359.67 ^c^ ± 11.59	1281.0 ^c^ ± 19.92
Infected + Amprolium	282.67 ± 7.09	373.00 ^c^ ± 8.62	443.33 ^b^ ± 8.62	455.00 ^b^ ± 10.81	1554.0 ^b^ ± 24.06
GSP2.5 + OZ50	289.67 ± 1.52	386.33 ^ab^ ± 4.72	422.67 ^c^ ± 4.72	458.00 ^b^ ± 10.53	1558.0 ^b^ ± 14.97
GSP5 + OZ50	288.67 ± 4.72	379.33 ^ab^ ± 3.05	412.67 ^c^ ± 3.05	452.00 ^b^ ± 7.63	1533.0 ^b^ ± 12.34
*p*-value	0.128	≤0.01	≤0.01	≤0.01	≤0.01

Mean values bearing different superscripts (a, b, c) in the column differ significantly (*p* < 0.05); GSP: grape seed powder; OZ: organic Zn.

**Table 4:** Mean ± SE of feed conversion ratio of grape seed powder and organic Zn supplemented birds challenged with *Eimeria tenella*.

Groups	2nd Week	3rd Week	4th Week	5th Week	Overall Mean
Negative control	1.44 ± 0.39	1.60 ^b^ ± 0.01	1.53 ^c^ ± 0.14	1.78 ^d^ ± 0.02	1.62 ^d^ ± 0.02
Positive control	1.38 ± 0.04	1.67 ^a^ ± 0.02	2.15 ^a^ ± 0.01	2.21 ^a^ ± 0.03	1.87 ^a^ ± 0.01
Infected + Amprolium	1.37 ± 5.77	1.65 ^a^ ± 0.02	1.62 ^c^ ± 5.77	1.89 ^b^ ± 1.01	1.74 ^c^ ± 5.77
GSP2.5 + OZ50	1.37 ± 0.01	1.64 ^a^ ± 0.01	1.75 ^b^ ± 0.034	1.98 ^b^ ± 0.06	1.75 ^bc^ ± 0.02
GSP5 + OZ50	1.37 ± 0.01	1.65 ^a^ ± 0.01	1.78 ^b^ ± 0.01	1.98 ^b^ ± 0.01	1.78 ^b^ ± 0.01
*p*-value	0.35	≤0.01	≤0.01	≤0.01	≤0.01

Mean values bearing different superscripts (a, b, c, d) in the column differ significantly (*p* < 0.05); GSP: grape seed powder; OZ: organic zinc.

**Table 5:** Mean ± SE of dressing percentage of grape seeds powder and organic Zn of broilers challenged with *E. tenella.*

Group	Dressing Percentage
Negative control	68.55 ^a^ ± 1.19
Positive control	58.35 ^d^ ± 2.56
Infected + Amprolium	67.45 ^ab^ ± 0.72
GSP2.5 + OZ50	65.98 ^bc^ ± 0.35
GSP5 + OZ50	64.99 ^c^ ± 0.22
*p*-value	<0.001

Mean values bearing different superscripts (a, b, c, d) in the column differ significantly (*p* < 0.05); GSP: Grape seed powder; OZ: organic zinc.

**Table 6:** Mean ± SE of oocyst per gram of feces in grape seed powder and organic Zn supplemented birds in *E. tenella* challenged broilers at 7, 14, and 21 days post-infection (dpi).

Group	7 dpi	14 dpi	21 dpi
Negative control	0.00 ^d^ ± 0.00	0.00 ^d^ ±0.00	0.00 ^d^ ± 0.00
Positive control	705.00 ^a^ ± 25.00	833.33 ^a^ ± 33.29	285.00 ^a^ ± 20.00
Infected + Amprolium	370.00 ^c^ ± 15.00	391.67 ^c^ ± 15.27	120.00 ^c^ ± 22.91
GSP2.5 + OZ50	477.00 ^b^ ± 20.8	525.40 ^b^ ± 12.09	248.33 ^b^ ± 17.55
GSP5 + OZ50	498.33 ^b^ ± 31.22	541.67 ^b^ ± 41.96	223.33 ^b^ ± 47.25
*p*-value	≤0.01	≤0.01	≤0.01

Mean values bearing different superscripts (a, b, c, d) in the column differ significantly (*p* < 0.05); GSP: Grape seed powder; OZ: organic zinc.

**Table 7:** Lesion score of cecum of grape seed powder and organic zinc supplemented birds in coccidiosis challenged broilers.

Groups	Thickness of Ceca	Hemorrhages	Congestion
Negative control	0	0	0
Positive control	+++	+++	++
Infected + Amprolium	+	+	0
GSP2.5 + OZ50	++	++	+
GSP5 + OZ50	++	++	+

0 = no lesion, + = mild lesion, ++ = moderate lesion, +++ = severe lesion.

## Data Availability

Data presented in this study are available on fair request from the respective author.
